# Trends and Research Frontiers on Exclusive Breastfeeding Policy: A Bibliometric Analysis

**DOI:** 10.12688/f1000research.181875.2

**Published:** 2026-08-11

**Authors:** Yunita Simon, Veni Hadju, Citrakesumasari Dahlan, Muhammad Chaeroel Ansar

**Affiliations:** 1 Nutritional Science, Hasanuddin University School of Public Health, Makassar, South Sulawesi, 90245, Indonesia; 2 Government Science, Hasanuddin University Faculty of Social and Political Sciences, Makassar, South Sulawesi, 90245, Indonesia

**Keywords:** Exclusive breastfeeding, Breastfeeding policy, Bibliometric analysis, Maternal and child health, Global health

## Abstract

**Background:**

Exclusive breastfeeding (EBF) is vital for child survival, growth, and development, yet its promotion and protection remain challenging across diverse policy and health system contexts. Over the past decade, research on EBF policy has increased alongside global health priorities and emerging crises. This study examines publication trends, key sources and authors, collaboration patterns, and thematic evolution in EBF policy research from 2016 to 2025 using bibliometric analysis.

**Methods:**

A bibliometric analysis was conducted using Scopus data retrieved with the query “exclusive breastfeeding” and “policy” for publications from 2016–2025. A total of 449 documents were analyzed based on publication trends, influential journals and authors, citation impact, collaboration networks, keyword co-occurrence, and thematic evolution.

**Results:**

EBF policy research showed a consistent annual growth rate of 18.57%, with publication peaks in 2020 and 2023, partly linked to the COVID-19 pandemic. The International Breastfeeding Journal and Maternal and Child Nutrition were the leading journals, while Pérez-Escamilla, Agho, and Ogbo were among the most influential authors. International collaboration increased, although contributions from low- and middle-income countries remained limited. Research themes evolved from child health and biomedical determinants (2016–2019) to broader issues, including maternal factors, workplace support, nutrition policy, and health system resilience, with COVID-19-related challenges emerging after 2020.

**Conclusions:**

EBF policy research has shifted toward a more holistic and system-oriented perspective. Strengthening policy frameworks, health systems, and equitable support mechanisms is essential to sustain progress in EBF promotion and protection.

## 1. Introduction

Exclusive breastfeeding (EBF) during the first six months of life is widely recognized as a critical intervention to improve infant and maternal health outcomes. The World Health Organization (WHO) and UNICEF have consistently recommended exclusive breastfeeding as a global standard for infant feeding, citing its protective effects against infections, malnutrition, and long-term non-communicable diseases ( Woolley & Wyver, 2020). Beyond health benefits, exclusive breastfeeding also contributes to cognitive development, emotional bonding, and cost savings for families and health systems ( Chade et al., 2024). Despite these advantages, global adherence to exclusive breastfeeding remains suboptimal, particularly in low- and middle-income countries where structural and policy challenges persist ( Izumi et al., 2024; Leppäniemi et al., 2023).

The policy environment surrounding exclusive breastfeeding has become increasingly important in shaping maternal behavior and societal norms. Policies such as maternity leave entitlements, workplace lactation support, health system regulations, and marketing restrictions on breast milk substitutes play a central role in enabling or constraining mothers’ ability to practice EBF ( Pèrez-Escamilla et al., 2018). In recent years, governments and international organizations have intensified their efforts to implement comprehensive breastfeeding policies, but the effectiveness of these measures varies across regions and contexts ( Freire et al., 2020). This variation highlights the need for systematic research to map the global knowledge base on exclusive breastfeeding policies.

Over the past decade, research on exclusive breastfeeding policy has expanded significantly, driven by rising concerns about maternal employment, commercial influences of the infant formula industry, and the global pursuit of Sustainable Development Goals (SDGs) ( Walters et al., 2021). Scholarly attention has shifted not only to the health benefits but also to the socio-political and economic determinants that influence breastfeeding practices ( Navarro-Rosenblatt et al., 2023). These evolving research frontiers indicate a growing recognition that EBF is not merely a personal choice but a policy and governance issue requiring multisectoral interventions.

Bibliometric analysis offers a useful lens to trace how research on exclusive breastfeeding policy has evolved, identify leading contributors, and uncover emerging thematic clusters. Unlike narrative reviews, bibliometric approaches allow for a quantitative mapping of scientific production, citation networks, and keyword co-occurrence patterns, providing a comprehensive overview of the intellectual and conceptual structure of the field ( Izumi et al., 2024). This method is particularly relevant for exclusive breastfeeding research, where the intersection of health policy, nutrition science, and social determinants necessitates a multidisciplinary perspective ( Rohini et al., 2022).

Previous bibliometric studies in related domains, such as maternal and child health, nutrition policy, and global health governance, have revealed shifting trends in research emphasis and the emergence of cross-cutting themes ( Sweileh, 2022). However, to date, few bibliometric analyses have been dedicated specifically to the exclusive breastfeeding policy. This gap makes it challenging to systematically understand how the scientific community has approached the topic in the last decade, particularly about the balance between biomedical, social, and policy-related dimensions. A focused bibliometric study could therefore illuminate both the strengths and limitations of existing scholarship.

Mapping the knowledge structure of exclusive breastfeeding policy research also holds practical value for policymakers, health advocates, and practitioners. By identifying influential publications, research institutions, and thematic hotspots, bibliometric analysis can guide future collaborations and policy dialogues ( Aria & Cuccurullo, 2017). For instance, understanding how research aligns with global policy frameworks such as the International Code of Marketing of Breast-milk Substitutes or the Baby-Friendly Hospital Initiative could highlight areas where evidence-based advocacy has been most effective or where gaps remain ( WHO, 2020).

Against this backdrop, this study conducts a bibliometric analysis of exclusive breastfeeding policy research published over the past decade. Specifically, it aims to examine publication trends, leading authors and institutions, influential journals, and thematic evolution in the literature. By doing so, this article seeks to identify research frontiers and potential directions for advancing both academic inquiry and evidence-informed policymaking on exclusive breastfeeding. In addressing these objectives, the study contributes to a more nuanced understanding of how exclusive breastfeeding policy has been conceptualized and prioritized within global scholarly discourse.

## 2. Materials and methods

### 2.1 Data source and search strategy

A bibliometric search was conducted using the Scopus database because of its broad coverage of peer-reviewed scientific literature. To improve the comprehensiveness of the search and reduce the likelihood of missing relevant publications, the search strategy included the core concept of exclusive breastfeeding and related policy-oriented terms. The search was performed in the title, abstract, and author keyword fields using the following Scopus search query:

(TITLE-ABS-KEY(“exclusive breastfeeding”) AND TITLE-ABS-KEY (policy)) OR TITLE-ABS-KEY(“breastfeeding policy”) OR TITLE-ABS-KEY(“exclusive breastfeeding policy”) OR TITLE-ABS-KEY(“infant feeding policy”)

The search was restricted to publications in English and to the period from 2016 to 2025 using the following filters: *LIMIT-TO (LANGUAGE, “English”), PUBYEAR > 2015, and PUBYEAR < 2026*. The complete search strategy was designed to capture publications addressing exclusive breastfeeding in relation to policy, as well as studies explicitly using related breastfeeding and infant-feeding policy terminology. The full search string and filtering criteria were retained to facilitate reproducibility of the bibliometric search ( Ansar et al., 2026a, b).

The initial search identified 9,495 records. After application of the predefined publication-year and language filters, 6,670 records remained for subsequent screening. These records were screened based on their titles, abstracts, and keywords to identify publications relevant to exclusive breastfeeding policy research, resulting in 616 potentially relevant records. The 616 records were subsequently assessed for eligibility based on their relevance to the study scope and open-access availability, resulting in 449 records for the final bibliometric analysis.

**Figure 1.**
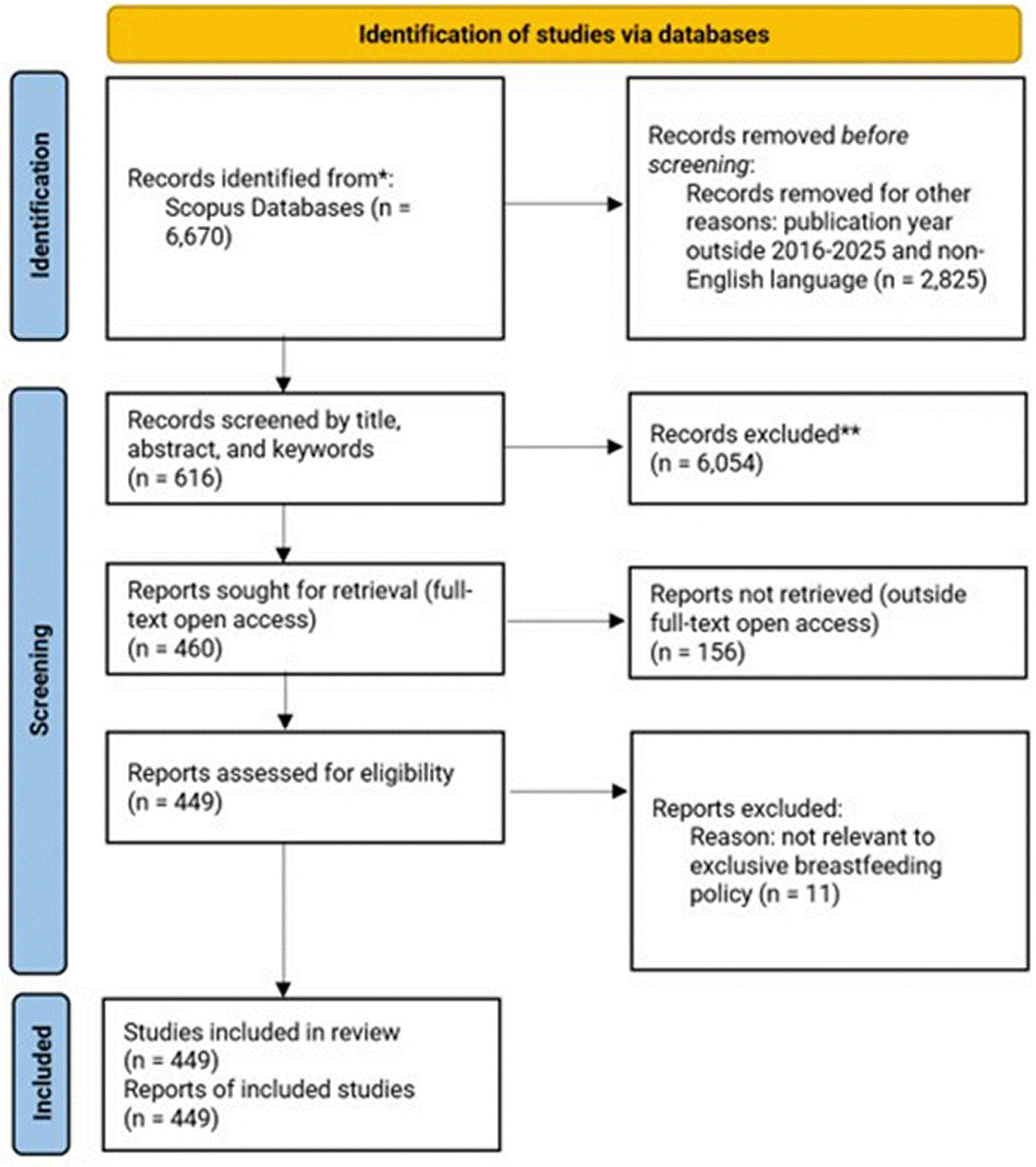
PRISMA flow diagram of the article search and screening for data extraction.

The study selection process was presented using a PRISMA flow diagram solely to provide transparency in the identification, screening, eligibility assessment, and inclusion of bibliographic records. PRISMA was not assessing the methodological quality or risk of bias of individual studies. During the screening stage, 6,054 records were excluded because they were not sufficiently relevant to the scope of exclusive breastfeeding policy research. Of the 616 records assessed for eligibility, 167 were excluded because they did not meet the predefined scope and/or open-access criteria. Consequently, 449 records were included in the final bibliometric dataset ( Figure 1).

### 2.2 Inclusion and exclusion criteria

All bibliographic records retrieved from Scopus that met the predefined search strategy and were relevant to exclusive breastfeeding policy research were considered eligible for inclusion in the bibliometric analysis. Unlike a systematic review that may restrict eligibility to specific study designs, this bibliometric study included different document types indexed in Scopus in order to provide a comprehensive mapping of the research landscape. Accordingly, articles, short surveys, conference papers, editorials, reviews, and errata were retained when they met the predefined eligibility criteria. Open-access availability was included as an eligibility criterion to ensure that the publications included in the final dataset could be accessed and independently verified during the screening and eligibility assessment process. This criterion was adopted as a pragmatic methodological decision to facilitate consistent assessment of publication relevance and scope and to support transparency and reproducibility of the selection process. The open-access criterion was applied at the eligibility stage after initial screening rather than as a criterion for determining the scientific relevance of the publications.

Records were excluded when their title, abstract, or keywords indicated that they were not relevant to exclusive breastfeeding or its policy-related context. Publications that did not meet the predefined publication period or language criteria described in Section 2.1, as well as records that did not meet the predefined open-access criterion, were excluded. The final eligible dataset comprised 449 records and was subsequently used for bibliometric analysis. The inclusion of multiple document types was intended to capture the breadth and development of scholarly communication related to exclusive breastfeeding policy rather than to evaluate the methodological quality or effectiveness of individual studies. Because restricting the dataset to open-access publications may exclude relevant non-open-access research, the potential for selection bias associated with the open-access criterion was considered as a limitation of the study.

### 2.3 Data extraction

Bibliographic data were exported from Scopus in formats compatible with bibliometric software (.csv for R version 4.5.1). Extracted variables included authors, publication year, journal title, institutional and country affiliations, document type, citation counts, abstracts, and author keywords. Following full-text retrieval, the dataset was refined to include 449 eligible documents that met all inclusion criteria and were relevant to exclusive breastfeeding policy research.

### 2.4 Quality assessment of selected studies

Given the bibliometric nature of this study, a conventional methodological quality or risk-of-bias assessment was not performed. Instead, quality control was ensured through strict inclusion criteria, reliance on peer-reviewed articles indexed in Scopus, and systematic screening following the PRISMA flow diagram. This approach is consistent with established bibliometric research practices, which focus on research output characteristics, collaboration structures, and thematic development rather than study-level methodological quality.

### 2.5 Data analysis

Bibliometric analyses were conducted using CiteSpace and the Bibliometrix package in R version 4.5.1. These software tools were combined to analyze the final dataset and examine publication trends, influential sources and authors, collaboration patterns, citation relationships, keyword co-occurrence, and thematic evolution of exclusive breastfeeding policy research from 2016 to 2025. Descriptive indicators such as annual publication output and citation performance were used to assess research growth. Co-authorship analyses explored collaboration networks among authors, institutions, and countries. Keyword co-occurrence and thematic mapping analyses were employed to identify major research themes and their evolution over time. The results were synthesized to illustrate the intellectual structure and development trajectory of exclusive breastfeeding policy research.

## 3. Results and discussion

### 3.1 Descriptive analysis of publications

***3.1.1 Main information on exclusive breastfeeding (EBF) policy***

The bibliometric dataset on EBF policy covers publications from 2016 to 2025, yielding a total of 449 documents across 177 different sources including journals, books, and conference proceedings ( Table 1). The annual growth rate of 18.57% reflects a steady expansion of scholarly interest in this field. On average, the documents are relatively recent, with an average age of 4.33 years, indicating that exclusive breastfeeding policy has emerged as a more prominent research focus within the past decade. This pattern is consistent with global health and policy agendas that have increasingly emphasized breastfeeding as part of the Sustainable Development Goals ( Requejo et al., 2015).

**Table 1.** Main information on the exclusive breastfeeding policy.

**MAIN INFORMATION ABOUT DATA**	
**Timespan**	**2016:2025**
Sources (Journals, Books, etc.)	177
Documents	449
Annual Growth Rate %	18.57
Document Average Age	4.33
Average citations per doc	15.34
References	45890
**DOCUMENT CONTENTS**	
Keywords Plus (ID)	1890
Author’s Keywords (DE)	1080
**AUTHORS**	
Authors	3485
Authors of single-authored docs	15
**AUTHORS COLLABORATION**	
Single-authored docs	15
Co-Authors per Doc	8.87
International co-authorships %	43.88
**DOCUMENT TYPES**	
Article	380
Conference paper	4
Editorial	2
Erratum	1
Review	1
Short survey	61

In terms of research visibility, the documents have received an average of 15.34 citations per publication, suggesting that studies in this domain are gaining traction and contributing significantly to wider academic discussions. While the absence of listed references in the dataset may reflect database extraction limitations, the presence of 1,890 Keywords Plus and 1,080 author’s keywords highlights the conceptual richness and multidimensional scope of this literature. The diversity of keywords suggests that exclusive breastfeeding policy research is not confined to health and nutrition but intersects with themes such as governance, maternal employment, and marketing regulation, aligning with previous findings on the multidisciplinarity of breastfeeding research ( Piwoz & Huffman, 2015; Baker et al., 2021).

The dataset also demonstrates broad author participation, with 3,485 contributing authors. Among these, 15 were single-authored publications, resulting in 15 single-authored documents overall. However, the majority of publications involve collaboration, as indicated by the average of 8.87 co-authors per document and an international co-authorship rate of 43.88%. This level of collaboration suggests that exclusive breastfeeding policy is increasingly recognized as a global issue requiring cross-country comparative studies and international partnerships. The rising trend of international collaboration mirrors similar patterns in maternal and child health research, where global networks have been crucial in advancing evidence-based policies ( Sweileh, 2025).

Regarding document types, most outputs are journal articles (380), followed by reviews (61), conference papers (4), and smaller numbers of editorials, erratum, and short surveys. The dominance of journal articles indicates that the field is primarily driven by peer-reviewed empirical and conceptual contributions, while the presence of review articles suggests an effort to synthesize and evaluate the growing body of evidence. The relatively small number of editorials and erratum may imply that exclusive breastfeeding policy is still a developing domain where journals serve as the primary medium for scholarly exchange. This distribution reflects broader trends in health policy research, where journal articles are the main vehicle for both academic visibility and policy influence ( Donthu et al., 2022).

***3.1.2 Publication trends***

The annual distribution of publications on EBF policy from 2016 to 2025 demonstrates a clear overall growth trend, despite some year-to-year fluctuations. The field began with a relatively small number of publications in 2016 (19 articles), and a temporary decline was observed in 2017 (16 articles), indicating the early development of EBF policy as a distinct area of scholarly attention. Furthermore, publications increased modestly in 2018 (31 articles) and afterwards till 2025, with over 40 records, suggesting renewed research interest and growing policy engagement.

The highest number of publications was recorded in 2025 (88 articles), reflecting intensified global attention to breastfeeding policy issues. This growth period coincides with increasing policy discussions surrounding maternal protection, workplace breastfeeding support, and regulation of breast milk substitute marketing, as well as heightened concern for maternal and child nutrition during and after the COVID-19 pandemic. The trend indicates that EBF policy research has gained sustained momentum over the past decade, establishing itself as an increasingly important area of public health and policy scholarship.

***3.1.3 Most relevant sources***

The analysis of the most relevant sources identifies the key journals contributing to the dissemination of EBF policy research over the study period. The International Breastfeeding Journal is the leading outlet, publishing 61 articles, reflecting its strong specialization in breastfeeding promotion, maternal and child health, and policy-related issues. This prominence is consistent with its role as a central platform for global discussions on breastfeeding practices and policy implementation, including the International Code of Marketing of Breast-milk Substitutes ( Rollins et al., 2016). The Maternal and Child Nutrition journal follows with 31 articles, underscoring its influence in advancing research on maternal and child nutrition and related policy interventions.

Several multidisciplinary and open-access journals also make substantial contributions to the field. PLOS ONE (21 articles) and BMC Public Health (13 articles) provide broader public health perspectives, facilitating the integration of breastfeeding policy research into wider health and population-based discussions. Similarly, the International Journal of Environmental Research and Public Health (12 articles) highlights the increasing intersection between breastfeeding, environmental health, and sustainable development agendas ( Emmott, 2023). Additional specialized outlets, including BMC Pregnancy and Childbirth (11 articles), Nutrients (9 articles), BMC Pediatrics (7 articles), BMJ Open (6 articles), and Frontiers in Nutrition (6 articles), further demonstrate the multidisciplinary nature of EBF policy research. The distribution of sources indicates that while the field remains strongly anchored in specialized maternal and child health journals, it is increasingly disseminated through interdisciplinary and open-access platforms, reflecting the complex and multi-sectoral character of exclusive breastfeeding policy research.

***3.1.4 Most relevant authors***

The author-level analysis indicates that research on EBF policy over the past decade has been shaped by several highly productive scholars. Rafael Pérez-Escamilla stands out as the most prolific author, contributing 10 publications (fractionalized 2.28). His extensive work is well recognized in the field, particularly on the impact of breastfeeding policies, the Baby-Friendly Hospital Initiative (BFHI), and the role of socio-political contexts in shaping maternal and child health outcomes ( Pèrez-Escamilla et al., 2019). His prominence underscores the centrality of evidence-based advocacy in strengthening global breastfeeding programs.

Following closely are Kingsley E. Agho and Felix A. Ogbo, each with 8 publications (fractionalized 1.49). Both have made significant contributions to the understanding of socio-demographic determinants of breastfeeding practices, particularly within the African and Asia-Pacific regions. Their studies highlight disparities in breastfeeding uptake and explore how maternal education, employment, and health systems influence EBF practices ( Ogbo et al., 2017). Such findings are crucial in informing context-specific policy strategies to improve breastfeeding outcomes in low- and middle-income countries.

Other notable contributors include Al-Jawadleh AE, Buccini GDS, and Page AN, each with 6 publications, as well as Kimani-Murage EW, Nguyen TT, and Unar-Munguía M with 5 publications each. Their research spans diverse issues, from marketing of breastmilk substitutes and community-level interventions ( Hromi-Fiedler et al., 2019) to structural barriers facing women in urban Africa ( Kimani-Murage et al., 2015). The presence of Ahmed TJ (4 publications) and others indicates that EBF policy research is characterized by a collaborative and multidisciplinary authorship pattern, supported by the average of over five co-authors per document in this dataset.

The prominence of these authors reflects a research community that is both globally distributed and highly collaborative. The literature shows that leading voices such as Pérez-Escamilla and Agho have played pivotal roles in linking empirical findings with advocacy and policymaking, thereby shaping the global breastfeeding agenda. The diverse geographic focus of these scholars further emphasizes that exclusive breastfeeding policy is not a uniform challenge, but one deeply embedded in cultural, economic, and political realities across regions.

### 3.2 Citation and impact analysis

***3.2.1 Most cited documents***

The analysis of the most cited documents highlights key studies that have shaped EBF policy research over the past decade ( Table 2). Highly cited publications mainly focus on determinants of EBF, policy implementation, and population-level patterns, emphasizing the applied and policy-oriented nature of the field. The most cited study by Kimani-Murage et al. (2015) examines barriers to implementing WHO breastfeeding recommendations in urban poor settings, reflecting strong scholarly attention to structural challenges in low- and middle-income countries. Other influential works, including Pérez-Escamilla and Buccini ( Pérez-Escamilla et al., 2019), have stimulated global debate on the relevance and feasibility of the six-month exclusive breastfeeding recommendation, while studies by Ogbo, Agho, and Page ( Ogbo et al., 2015, 2019) provide large-scale evidence on socio-demographic and health system determinants of EBF.

**Table 2.** Most cited documents in exclusive breastfeeding policy.

Author	Year	TI	SO	DOI	TC
Kimani-Murage EW	2015	Factors affecting actualisation of the who breastfeeding recommendations in urban poor settings in Kenya	Maternal and child nutrition	10.1111/mcn.12161	110
Pèrez-Escamilla R	2019	Perspective: should exclusive breastfeeding still be recommended for 6 months?	Advances in nutrition	10.1093/advances/nmz039	76
Buccini GDS	2019	Perspective: should exclusive breastfeeding still be recommended for 6 months?	Advances in nutrition	10.1093/advances/nmz039	76
Ogbo Fa	2019	Regional prevalence and determinants of exclusive breastfeeding in india	International breastfeeding journal	10.1186/s13006-019-0214-0	62
Agho Ke	2019	Regional prevalence and determinants of exclusive breastfeeding in india	International breastfeeding journal	10.1186/s13006-019-0214-0	62
Page An	2019	Regional prevalence and determinants of exclusive breastfeeding in india	International breastfeeding journal	10.1186/s13006-019-0214-0	62
Pèrez-Escamilla R	2018	Becoming breastfeeding friendly index: development and application for scaling-up breastfeeding programmes globally	Maternal and child nutrition	10.1111/mcn.12596	58
Ogbo Fa	2015	Determinants of suboptimal breastfeeding practices in nigeria: evidence from the 2008 demographic and health survey	Bmc public health	10.1186/s12889-015-1595-7	58
Agho Ke	2015	Determinants of suboptimal breastfeeding practices in nigeria: evidence from the 2008 demographic and health survey	Bmc public health	10.1186/s12889-015-1595-7	58
Buccini GDS	2018	Becoming breastfeeding friendly index: development and application for scaling-up breastfeeding programmes globally	Maternal and child nutrition	10.1111/mcn.12596	58

Additional highly cited contributions, such as the Becoming Breastfeeding Friendly (BBF) Index developed by Pérez-Escamilla and Buccini ( Pérez-Escamilla et al., 2018), offer important policy frameworks for assessing national readiness to scale up breastfeeding programs. Studies on suboptimal breastfeeding practices in Nigeria further highlight the influence of socio-economic and health service factors on EBF outcomes. Overall, citation patterns indicate that EBF policy research is strongly grounded in population-based evidence and policy evaluation, with a growing focus on implementation challenges, equity, and scalability. This trend reflects the field’s shift toward a more policy- and systems-oriented approach to improving breastfeeding practices globally.

***3.2.2 Source impact***

The impact of sources on EBF policy research can be assessed using bibliometric indicators such as the h-index, g-index, m-index, and total citations (TC) ( Table 3). Among the sources, the International Breastfeeding Journal shows the highest influence, reflecting both a high volume of publications and sustained citation impact over time, and positioning it as a central platform for breastfeeding research and policy discussions ( Rollins et al., 2016). This is followed by Maternal and Child Nutrition, which also demonstrates strong and consistent scholarly influence. Other specialized journals, including Breastfeeding Medicine and the Journal of Human Lactation, further highlight the important role of discipline-specific outlets in translating clinical and research evidence into policy-relevant knowledge ( Pèrez-Escamilla et al., 2019).

**Table 3.** Source impact based on h-index, g-index, and m-index.

Source	h_index	g_index	m_index	TC
International Breastfeeding Journal	21	35	1.75	1379
Maternal and Child Nutrition	16	26	1.45	698
Breastfeeding Medicine	11	18	1.1	347
Journal of Human Lactation	11	16	1	296
PLOS One	10	16	0.83	276
BMC Public Health	9	13	0.81	203
Nutrients	8	9	0.67	196
International Journal of Environmental Research and Public Health	7	12	1	154
Maternal and Child Health Journal	7	12	0.58	325
BMC Pediatrics	6	7	0.75	141

In addition to specialized journals, multidisciplinary outlets such as PLOS ONE and BMC Public Health contribute to expanding the reach of EBF policy research to broader scientific audiences, enhancing its visibility and cross-disciplinary relevance. Similarly, journals like Nutrients and the International Journal of Environmental Research and Public Health reflect the integration of breastfeeding within wider public health and nutrition contexts ( Pérez-Escamilla et al., 2023). The findings reveal a dual pattern in which specialized journals drive citation impact, while multidisciplinary platforms amplify dissemination, ensuring both scientific rigor and broader policy influence ( Sinha et al., 2015).

***3.2.3 Author impact***

The author impact analysis highlights key scholars who have shaped EBF policy research over the past decade. Rafael Pérez-Escamilla emerges as the most influential author, with strong bibliometric indicators and a substantial number of citations, reflecting his leading role in breastfeeding research, particularly in relation to the Baby-Friendly Hospital Initiative (BFHI), global policy frameworks, and social determinants of maternal and child health ( Pèrez-Escamilla et al., 2019). Kingsley E. Agho and Felix A. Ogbo follow closely, both demonstrating significant impact through large-scale epidemiological studies on breastfeeding practices, especially in low- and middle-income countries, where they identify important socioeconomic and health system determinants of EBF ( Ogbo et al., 2017).

Other contributors, including Al-Jawadleh AE, Page AN, Buccini GDS, and Kimani-Murage EW, further illustrate the diversity of research areas, ranging from structural barriers and policy implementation to community-level challenges and formula marketing regulation ( Kimani-Murage et al., 2015). Overall, the findings indicate that EBF policy research is driven by a relatively small but highly influential group of scholars whose work integrates empirical evidence with policy advocacy. Their sustained contributions and collaborations play a crucial role in translating research into global guidelines and strengthening the overall impact of breastfeeding policies ( Victora et al., 2016).

### 3.3 Intellectual structure of breastfeeding policy

***3.3.1 Co-citation analysis***

**Figure 2.**
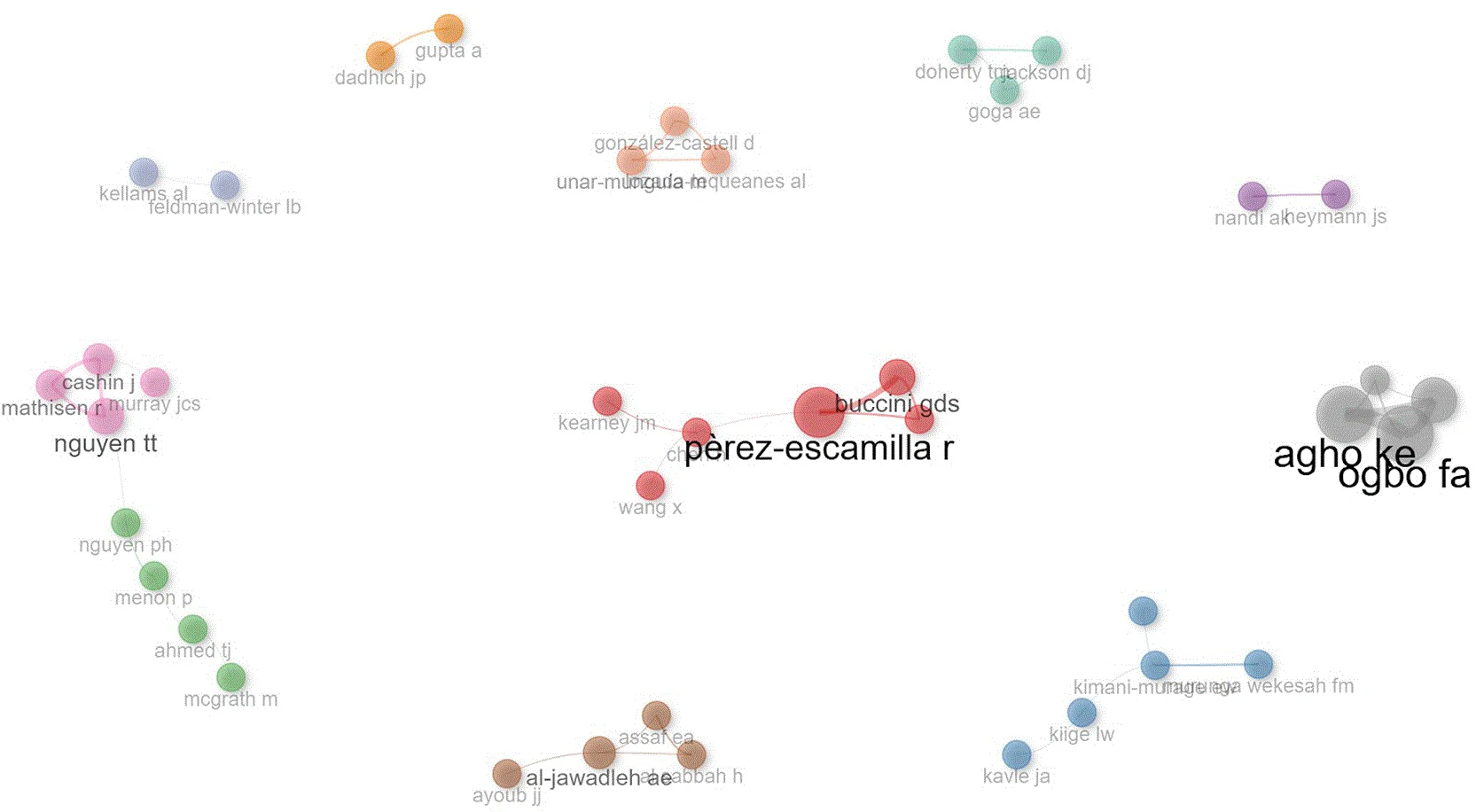
Author co-citation network map on exclusive breastfeeding policy.

The co-citation analysis reveals the intellectual structure of EBF policy research by identifying frequently co-cited authors and their scholarly clusters ( Figure 2). Two main clusters emerge. Cluster 1 is centered on Pérez-Escamilla R, who shows high influence and connectivity, alongside authors such as Buccini GDS, Chen H, and others. This group focuses on breastfeeding promotion, hospital-based initiatives, and regulatory frameworks such as the International Code of Marketing of Breast-milk Substitutes, emphasizing the importance of institutional and policy-level drivers in shaping EBF outcomes ( Pèrez-Escamilla et al., 2019).

Cluster 2 is led by Kimani-Murage EW, who plays a central role in research on contextual and community-level determinants of breastfeeding. Together with authors such as Ickes SB and Kavle JA, this cluster examines socio-cultural, economic, and health system factors affecting EBF practices, particularly in low- and middle-income countries ( Kimani-Murage et al., 2015). The analysis highlights two complementary research streams: one focused on global policy and advocacy, and the other on local implementation and contextual realities. The integration of these perspectives is essential for advancing effective and context-sensitive breastfeeding policies ( Victora et al., 2016).

***3.3.2 Collaboration networks***

**Figure 3.**
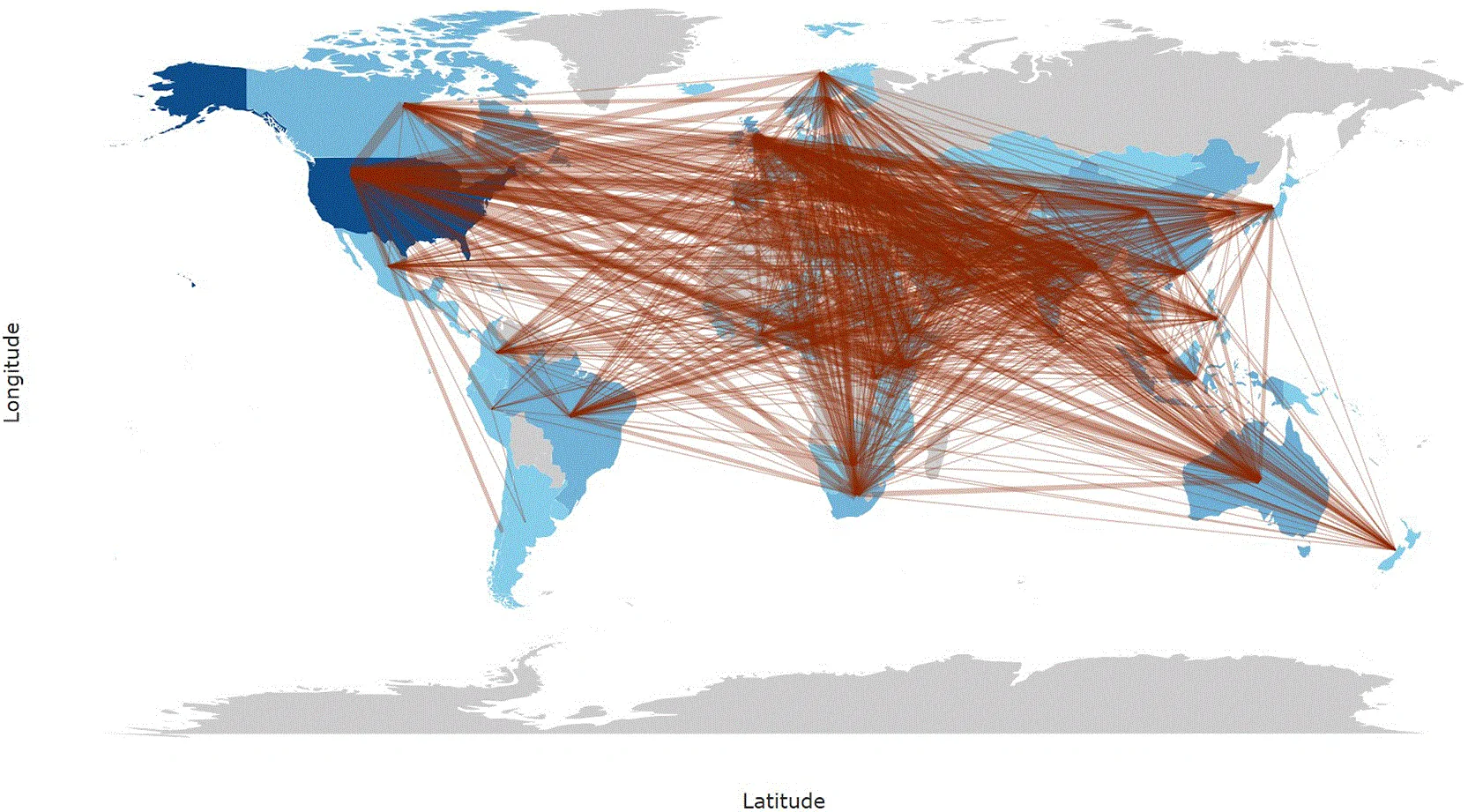
Countries' collaboration network on exclusive breastfeeding policy.

The collaboration network analysis highlights the importance of transnational partnerships in EBF policy research ( Figure 3). Argentina emerges as a key hub, maintaining strong research connections with countries such as Bahrain, Cyprus, Finland, Estonia, and Bulgaria, indicating active co-authorship and participation in multinational initiatives on maternal and child health ( Victora et al., 2016). The country also shows notable collaboration with Congo, reflecting the growing role of South–South partnerships in addressing shared challenges among low- and middle-income countries. However, weaker or negative collaboration patterns with countries such as Gambia and Guatemala suggest uneven research connectivity, likely influenced by differences in funding, academic networks, and policy priorities.

These findings confirm that international collaboration plays a crucial role in enhancing research visibility, impact, and policy relevance in EBF studies. Cross-country partnerships not only increase citation potential but also support knowledge exchange and policy transfer across diverse contexts ( Moed et al., 2005). In line with previous studies, global initiatives and coalitions in breastfeeding promotion rely heavily on such collaborations to strengthen advocacy and monitor progress. The central role of Argentina further highlights the growing contribution of Latin America in bridging collaborations between high-income countries and other LMICs.

### 3.4 Conceptual structure and thematic evolution

***3.4.1 Keyword co-occurrence***

**Figure 4.**
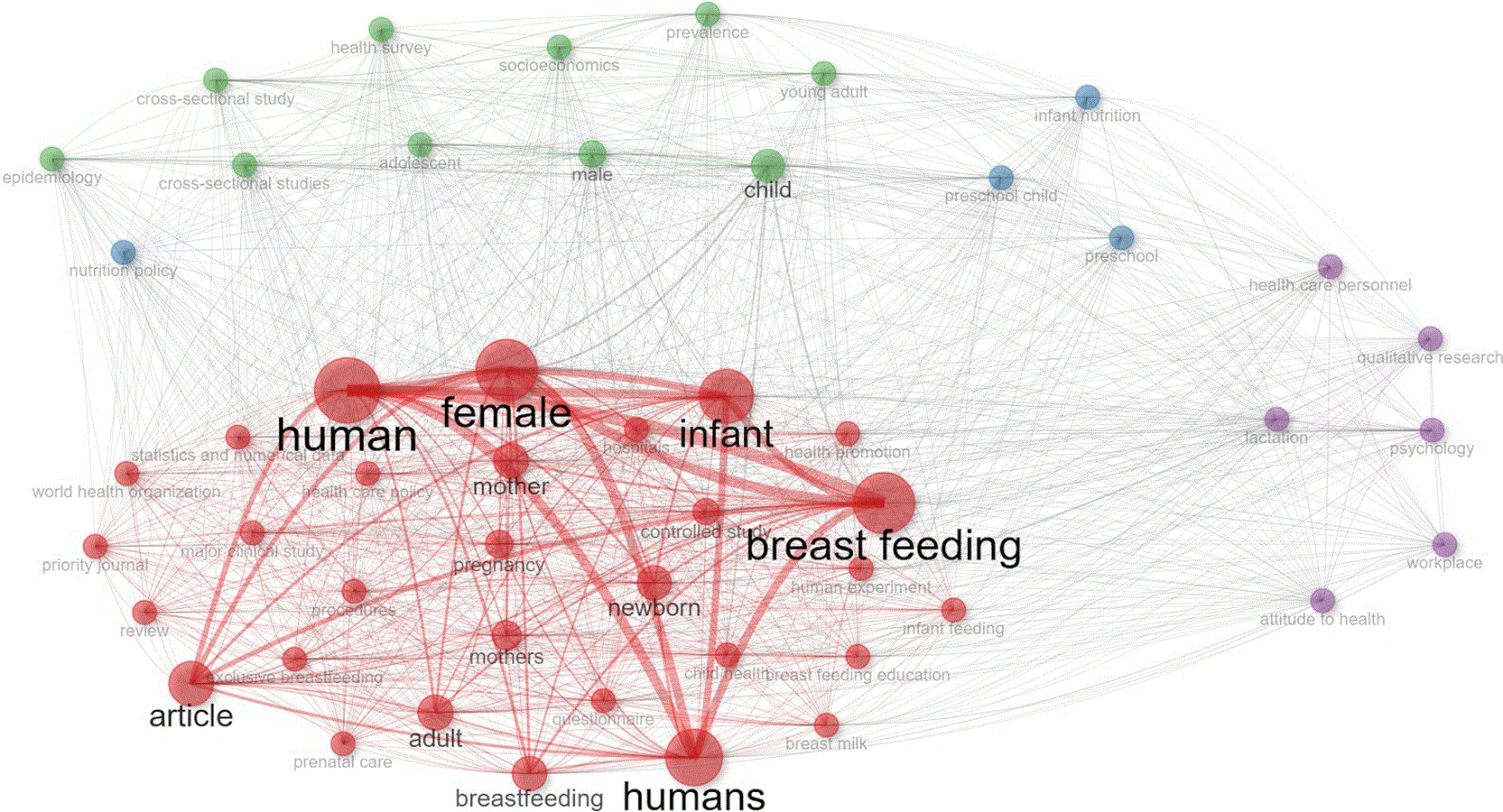
Keyword co-occurrence network map on exclusive breastfeeding policy.

The keyword co-occurrence analysis highlights the main thematic structure of EBF research, with dominant terms such as “human,” “breast feeding,” and “female” acting as central nodes within a single large cluster ( Figure 4). These keywords, along with related terms like “infant,” “newborn,” and “mother,” indicate that the literature strongly emphasizes the maternal–child health relationship as the core focus of breastfeeding research. High betweenness values suggest that these terms connect various subtopics, reinforcing the central role of breastfeeding as both a biological and public health concern. This pattern shows that much of the research remains grounded in biomedical and clinical perspectives, where maternal and infant health outcomes are the primary focus ( Wang et al., 2021).

From a broader perspective, this trend aligns with global research patterns that prioritize nutritional, developmental, and disease prevention outcomes in breastfeeding studies. However, it also suggests a relative underrepresentation of broader determinants such as workplace support, social environments, and structural inequalities. Previous studies highlight the need to expand the focus beyond individual-level factors to include systemic and policy-related influences on breastfeeding practices. Therefore, while the keyword network demonstrates strong research density around core biomedical themes, it also reveals important gaps in addressing social, economic, and policy dimensions of EBF ( Wang et al., 2021).

***3.4.2 Thematic evolution***

**Figure 5.**
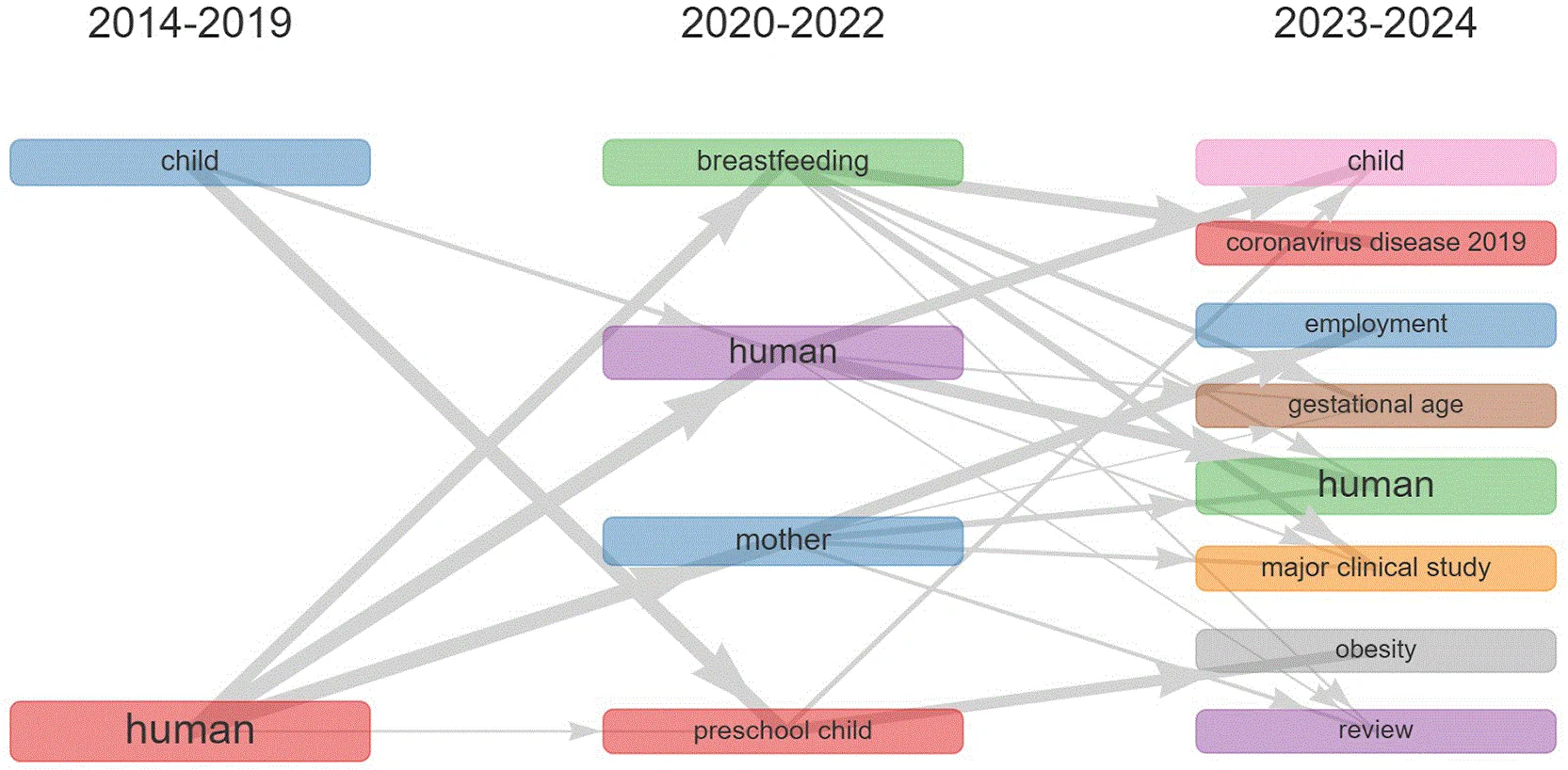
Thematic evolution of research themes across time periods.

The thematic evolution analysis shows a clear shift in exclusive breastfeeding (EBF) research priorities over time, reflecting increasing thematic specificity and broader policy orientation ( Figure 5). During 2016–2019, research was dominated by general themes such as child and human, focusing on infant health, child development, nutritional status, and socio-demographic risk factors. In 2020–2022, these broad themes evolved into more focused clusters, including breastfeeding, mother, and preschool child. This transition reflects a growing emphasis on maternal determinants, workplace and social support, nutrition policy, stunting, and health system interventions, in line with global nutrition agendas and the Sustainable Development Goals.

In the most recent period (2023–2024), thematic evolution was strongly influenced by global disruptions, particularly the COVID-19 pandemic. Research attention shifted toward hospital-based practices, maternal health services, and adaptations of breastfeeding support policies such as the Baby-Friendly Hospital Initiative. At the same time, continuity in core themes related to maternal and child factors persisted. Overall, the thematic evolution highlights a progression from predominantly biomedical and child-centered perspectives toward more integrated approaches that incorporate policy, health systems, and social environments, underscoring the increasing complexity and maturity of EBF research.

## 4. Conclusion

This bibliometric analysis provides an overview of the development of exclusive breastfeeding (EBF) policy research from 2016 to 2025, highlighting trends in publications, key sources and authors, collaboration patterns, and thematic evolution. The findings show a steady increase in research output, particularly after 2019, reflecting growing global recognition of EBF as a public health priority and aligning with major initiatives such as the Sustainable Development Goals and emerging challenges like the COVID-19 pandemic. The results also emphasize the important role of specialized journals, influential authors, and expanding international collaborations in advancing the field, alongside a shift from predominantly clinical perspectives toward broader policy- and systems-oriented approaches. EBF policy research demonstrates increasing maturity and integration into global nutrition and child health agendas, with future directions needing to focus on equity, health system strengthening, and enhanced collaboration, particularly in low- and middle-income countries, to achieve global breastfeeding targets.

## Data citation

Ansar, M. C., Simon, Y., Hadju, V., & Citrakesumasari, D. (2026). PRISMA 2020 for “Trends and Research Frontiers on Exclusive Breastfeeding Policy: A Bibliometric Analysis”. [Dataset]. Zenodo. https://doi.org/10.5281/zenodo.19943811.

Ansar, M. C., Yunita, S., Hadju, V., & Citrakesumasari, D. (2026). Scopus bibliographic dataset on breastfeeding policy between 2016 and 2025 [Data set]. Zenodo. https://doi.org/10.5281/zenodo.19943107.

## Data Availability

Zenodo: Scopus bibliographic dataset on breastfeeding policy between 2016 and 2025. https://doi.org/10.5281/zenodo.19943107. (Ansar, M. C., Yunita, S., Hadju, V., & Citrakesumasari, D., 2026). Zenodo: Scopus bibliographic dataset on breastfeeding policy between 2016 and 2025. https://doi.org/10.5281/zenodo.19943107. (Ansar, M. C., Yunita, S., Hadju, V., & Citrakesumasari, D., 2026). Data are available under the terms of the Creative Commons Attribution 4.0 International license (CC-BY 4.0). This project contains the following extended data:
- [Dataset] (Collection of article data used for literature review). Zenodo: PRISMA abstracts/PRISMA/PRISMA-Scr checklist and flowchart for ‘Trends and Research Frontiers on Exclusive Breastfeeding Policy: A Bibliometric Analysis’. https://doi.org/10.5281/zenodo.19943811. (Ansar, M. C., Yunita, S., Hadju, V., & Citrakesumasari, D., 2026). Data are available under the terms of the Creative Commons Attribution 4.0 International license (CC-BY 4.0).
