# Supplementary material for: Trends and Research Frontiers on Exclusive Breastfeeding Policy: A Bibliometric Analysis
Source: F1000Res. 2026 Aug 11;15:792. Originally published 2026 May 25. [Version 2] doi: 10.12688/f1000research.181875.2 (PMC13562937; doi:10.12688/f1000research.181875.2)
Supplement: Peer review history

# Reviewer report: Reviewer response for version 2

**DOI:** 10.5256/f1000research.207827.r509913

**Published:** 2026-08-31

**Authors:** Zulfia Samiun

**Affiliations:** 1 Nursing Study Program, Universitas Muhammadiyah Makassar Fakultas Kedokteran, Makassar, South Sulawesi, Indonesia

The author has revised the manuscript based on feedback from previous reviewers.

Are the rationale for, and objectives of, the Systematic Review clearly stated?

Yes

Is the statistical analysis and its interpretation appropriate?

Partly

If this is a Living Systematic Review, is the ‘living’ method appropriate and is the search schedule clearly defined and justified? (‘Living Systematic Review’ or a variation of this term should be included in the title.)

Partly

Are sufficient details of the methods and analysis provided to allow replication by others?

Partly

Are the conclusions drawn adequately supported by the results presented in the review?

Partly

Reviewer Expertise:

Maternity Nursing, Mother and Child Health,Breastfeeding

I confirm that I have read this submission and believe that I have an appropriate level of expertise to confirm that it is of an acceptable scientific standard.

# Reviewer report: Reviewer response for version 2

**DOI:** 10.5256/f1000research.207827.r510416

**Published:** 2026-08-20

**Authors:** Suci Megawati

**Affiliations:** 1 Universitas Negeri Surabaya, Surabaya, Indonesia

The manuscript provides a relevant and useful bibliometric overview of research on exclusive breastfeeding policy. The study is methodologically sound, the analyses are appropriate, and the conclusions are adequately supported by the findings. Detailed comments is below:

 1. The rationale is clearly presented, and the study objectives are relevant and appropriately aligned with the bibliometric approach. The research gap and contribution of the study are sufficiently explained.

 2. The methods provide sufficient information regarding the Scopus database, search strategy, eligibility criteria, screening process, and bibliometric procedures to allow replication. The use of Bibliometrix and CiteSpace is appropriate for the stated objectives.

 3. The bibliometric analyses, including publication trends, citation analysis, collaboration networks, keyword co-occurrence, and thematic evolution, are appropriate and adequately interpreted. The results are presented in a clear and logical manner.

 4. The conclusions are consistent with the results and adequately reflect the main findings of the study. The identified shift toward broader policy, health-system, and social dimensions is supported by the thematic analysis.

 5. The manuscript is not presented as a Living Systematic Review.

 I have no substantive concerns that require revision and therefore recommend acceptance for publication without revision.

Are the rationale for, and objectives of, the Systematic Review clearly stated?

Yes

Is the statistical analysis and its interpretation appropriate?

Yes

If this is a Living Systematic Review, is the ‘living’ method appropriate and is the search schedule clearly defined and justified? (‘Living Systematic Review’ or a variation of this term should be included in the title.)

Not applicable

Are sufficient details of the methods and analysis provided to allow replication by others?

Yes

Are the conclusions drawn adequately supported by the results presented in the review?

Yes

Reviewer Expertise:

Public Policy and Sustainable Policy

I confirm that I have read this submission and believe that I have an appropriate level of expertise to confirm that it is of an acceptable scientific standard.

# Reviewer report: Reviewer response for version 1

**DOI:** 10.5256/f1000research.200764.r499488

**Published:** 2026-07-20

**Authors:** Zulfia Samiun

**Affiliations:** 1 Nursing Study Program, Universitas Muhammadiyah Makassar Fakultas Kedokteran, Makassar, South Sulawesi, Indonesia

Thank you for the opportunity to review the manuscript entitled “Trends and Research Frontiers on Exclusive Breastfeeding Policy: A Bibliometric Analysis”. The article possesses scientiDic merit and has the potential to make a valuable contribution to mapping the development of research on exclusive breastfeeding policy. However, there are several important issues that require attention, particularly regarding the transparency of the bibliometric methodology, the consistency of results reporting, and the depth of the

 discussion. Addressing these aspects will signiDicantly enhance the manuscript’s credibility, reproducibility and scientiDic quality.

 1. The search queries used were very simple, so it is highly likely that many relevant articles were missed. As a bibliometric study, the search strategy must be truly comprehensive. Please explain why only those two queries were used.

 2. The author has not speciDied the type of software used. Reviewers need to know which software was used so that the research can be replicated. Please specify the type of software used in the manuscript.

 3. Inconsistency in the number of sources. The ‘Results’ section states 153 sources, whilst Table 1 shows 177 sources. Please rectify this inconsistency.

 4. In the ‘Methods’ section, the author states that only ‘original research articles’ were included, yet Table 1 shows the inclusion of reviews, editorials, conference papers and others. This appears to be contradictory. Please clarify whether only original research was used or whether all types of documents were included.

 5. The authors have used a PRISMA Dlow diagram. However, bibliometric analysis does not actually require PRISMA in the same way as a systematic review does. Even if they wish to use PRISMA, the authors need to explain that the diagram is used solely to ensure transparency in the literature selection process.

 During the screening stage, many articles were also eliminated according to PRISMA. The PRISMA Digure should explain where those 6,221 articles went. Similarly, for the other stages, there needs to be an explanation or justiDication for exclusion.

 6. In bibliometric analysis, there is actually no requirement for articles to be open access. Many bibliometric studies analyse Scopus metadata without accessing the full text. The authors need to explain why only open-access articles were included. How was this Dilter applied in Scopus? Does this not introduce selection bias?

 7. The years are stated as 2016–2025 in the ‘Methods’ section, but the ‘Conclusion’ section states 2016–2026. Please correct this.

 8. Inconsistency in the average document age. Narrative result: 5.69 years; Table 1: 4.33 years. Please correct this.

 9. There are several duplicate references; for example, Pérez-Escamilla 2019 appears twice, and Pérez-Escamilla 2018 also appears twice. Please check this again.

 10. Under ‘Data availability’, the authors state “No data associated with this article”, but a Zenodo dataset is available below this. This is confusing and needs to be clariDied.

Are the rationale for, and objectives of, the Systematic Review clearly stated?

Yes

Is the statistical analysis and its interpretation appropriate?

Partly

If this is a Living Systematic Review, is the ‘living’ method appropriate and is the search schedule clearly defined and justified? (‘Living Systematic Review’ or a variation of this term should be included in the title.)

Partly

Are sufficient details of the methods and analysis provided to allow replication by others?

Partly

Are the conclusions drawn adequately supported by the results presented in the review?

Partly

Reviewer Expertise:

Maternity Nursing, Mother and Child Health,Breastfeeding

I confirm that I have read this submission and believe that I have an appropriate level of expertise to confirm that it is of an acceptable scientific standard, however I have significant reservations, as outlined above.

# Response

**Published:** 2026-08-01

**Authors:** Muhammad Chaeroel Ansar

We sincerely thank the reviewer for the constructive and insightful comments. We have carefully revised the manuscript to address all of the concerns raised, particularly regarding the transparency and reproducibility of the bibliometric methodology, consistency of the reported results, and clarification of the study selection and data availability. Our detailed responses to each comment are provided below.

1. We revised the search strategy to improve comprehensiveness by incorporating broader policy-related terms, including “exclusive breastfeeding,” “breastfeeding policy,” “exclusive breastfeeding policy,” and “infant feeding policy.” The complete Scopus search query and filtering criteria are now explicitly reported to enhance transparency and reproducibility (Section 2.1, Data source and search strategy).
2. We specified the bibliometric software used for the analysis, namely CiteSpace and the Bibliometrix package in R version 4.5.1, and clarified their use for analyzing the bibliographic dataset (Section 2.5, *Data analysis*).
3. We corrected the number of sources in the Results section from 153 to 177 to ensure consistency with Table 1 (Section 3.1.1, *Main information*).
4. We revised the inclusion and exclusion criteria to clarify that the bibliometric dataset included multiple Scopus-indexed document types, including articles, short surveys, conference papers, editorials, reviews, and errata. The previous statement restricting the dataset to original research articles has been removed (Section 2.2, *Inclusion and exclusion criteria*).
5. We clarified that the PRISMA flow diagram was used solely to provide transparency in the identification, screening, eligibility assessment, and inclusion of bibliographic records, rather than as a systematic review framework. We also clarified the exclusion process at each stage, including the 6,054 records excluded during screening and the 167 records excluded during eligibility assessment, resulting in the final dataset of 449 records (Section 2.1 and Figure 1).
6. We clarified that open-access availability was used as an eligibility criterion as a pragmatic methodological decision to facilitate consistent access and independent verification of publications during screening and eligibility assessment. The open-access criterion was applied at the eligibility stage after initial screening. We also acknowledged the potential for selection bias arising from the exclusion of relevant non-open-access publications (Section 2.2, *Inclusion and exclusion criteria*).
7. We corrected the study period stated in the Conclusion from 2016–2026 to 2016–2025 to ensure consistency with the predefined study period described in the Methods section (Conclusion section).
8. We corrected the reported average document age from 5.69 to 4.33 years in the Results section to ensure consistency with the value reported in Table 1 (Section 3.1.1, *Main information*).
9. We reviewed the reference list and removed duplicate references, including the duplicated Pérez-Escamilla 2018 and 2019 entries, to ensure that each reference appears only once in the reference list.
10. We revised the Data Availability statement to clarify that the bibliographic dataset used in this study is publicly available in Zenodo. The dataset, *Scopus bibliographic dataset on breastfeeding policy between 2016 and 2025*, has also been included in the reference list with its DOI (Data Availability section).
